# Rare diseases in Germany – Developments in the status of medical care

**DOI:** 10.25646/11746

**Published:** 2023-12-13

**Authors:** Miriam Schlangen, Katharina Heuing

**Affiliations:** Office of the National Action League for People with Rare Diseases (NAMSE)

**Keywords:** RARE DISEASES, NAMSE, NATIONAL ACTION PLAN, CENTRE MODEL, PATIENT PATHWAYS, GERMANY, CRITICAL PATHWAYS PATIENT CARE

## Abstract

**Background:**

Rare diseases are a heterogeneous group of complex clinical patterns, which more often than not run a chronic course. The fact that they are rare complicates the provision of medical care for the specific diseases.

**Results:**

In the field of action titled ‘Care, Centres, Networks’ of its National Action Plan, the National Action League for People with Rare Diseases recommends the formation of a three-level, interconnected centre model. This form of care was investigated in two large research projects. It was shown that the time to diagnosis was markedly reduced. Commissioned by the Federal Ministry of Health, the expert report on the health status of people with rare diseases in Germany issued in 2023 concludes that the medical care provided to this group of people has improved markedly since the National Action Plan was introduced. The establishment of the Centres for Rare Diseases (ZSE, Zentren für Seltene Erkrankungen) is seen as the most important development. However, it is noted that there is still a lack of coordinated care provision pathways for referring patients to the appropriate facilities.

**Conclusion:**

The provision of care to people with rare diseases has improved upon the implementation of the measures from the National Action Plan. In a next step, care provision pathways must be established across sector boundaries. Challenges remain in the area of psychosocial care and the long-term securing of funding for these structures.

## 1. Introduction

In the European Union (EU), a disease is considered rare if it afflicts no more than 5 in 10,000 people. According to estimates, roughly four million people with a rare disease live just in Germany [1], and some 30 million people are thought to be afflicted in the entire EU. Rare diseases form a group of very different and, in most cases, complex clinical patterns. Most rare diseases run a chronic course and are associated with restricted health and/or a limited life expectancy as they often lead to symptoms becoming manifest as early as in childhood. Some 80 % of rare diseases are genetically determined or co-determined, and they are rarely curable.

Rare diseases have some special characteristics that render both care and research more difficult: even though there are many of them when added up, the number of people afflicted by a particular rare disease is low. Also, there are usually only a few experts available who can provide care to people with the respective rare disease and conduct research on the disease. Quality-assured care structures are therefore rare. People with rare diseases often go through years of an odyssey through the health care system – at least five years on average – before a definitive diagnosis is made, and this despite the fact that there has been rapid progress in diagnostic capabilities in recent years. Often, this means that therapeutic measures can be initiated only rather late, and diagnosis may come too late for some of the afflicted. To date, there are no firmly established structures in the German health care system that enable a specific diagnostic work-up and treatment by the respective experts. Often, there is no drug treatment available for rare diseases, mainly due to a lack of incentives for research and development.

In 2009, the Council of the European Union called on all member states to define and implement a strategy for improvement of the health scenario of people with rare diseases by the end of 2013 [2]. At the same time, the German Federal Ministry of Health (BMG) published a research report on the health care status of people with rare diseases in Germany and described prioritised fields of action as well as proposals for improvement and solution scenarios on the basis of scientific analyses [3].

**Figure 1:**
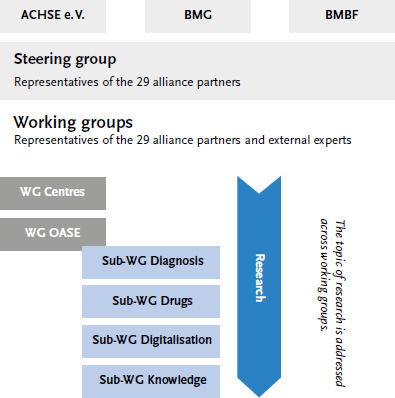
The National Action League for People with Rare Diseases (NAMSE) Source: own diagram

Subsequently, the National Action League for People with Rare Diseases (NAMSE) was founded in 2010 based on the joint initiative of the Federal Ministry of Health (BMG), Federal Ministry of Education and Research (BMBF) and Alliance of Chronic Rare Diseases (ACHSE e.V.), as well as 25 other alliance partners – all of them central and umbrella organisations of the major stakeholders in the health care system. Today, the League works in two working groups and four topic-specific sub-working groups; selected work results are discussed and approved by the steering group (for the work of NAMSE, see Figure 1).

In 2013, the League published a National Action Plan for People with Rare Diseases, in which a total of 52 measures in six fields of action were proposed to improve the health status of people with rare diseases. Chapter 3.1, field of action ‘Care, Centres, Networks’, is dedicated to the topic of care [4]. It proposes a three-tier centre model with three types of centres which do not differ in terms of the quality of care, but in the range of services offered. The three working levels are envisioned to be interconnected to each other. The implementation of the proposed measures is monitored and documented by the NAMSE office and was scientifically accompanied by a BMG-funded project (WB-NAPSE, 2015 – 2017) [5]. In 2023, a report commissioned by the BMG was published in which the current care status of people with rare diseases was analysed in comparison to 2009 [6].

The aim of the present article is to provide an overview of the developments in the care provided to people with rare diseases in Germany against the background of the work of NAMSE and other projects and initiatives.

**Infobox 1 Rare diseases**

A total of about 30,000 diseases are known worldwide, of which some 8,000 are rare diseases, also called ‘orphan diseases’. For example, children afflicted by Hutchinson-Gilford syndrome, also known as progeria, appear to age as if at a time-lapse pace. Only a few cases of the disease are known in Germany. The metabolic disease cystic fibrosis (CF) afflicts more than 8,000 people in Germany and is also a rare disease.

The European database for rare diseases, Orphanet (www.orpha.net), provides information on numerous rare diseases and medicines. The Care Atlas for Rare Diseases (se-atlas, www.se-atlas.de) provides an overview of care options for rare diseases in Germany. Information on rare diseases and patient associations is made available by an umbrella organisation called Alliance for Chronic Rare Diseases (ACHSE, www.achse-online.de).

**Infobox 2 Alliance partners of NAMSE**

► ACHSE e.V. Alliance of Chronic Rare Diseases
► Working Group of the Supreme State Health Authorities (AOLG) – represented by the respective chair state
► Association of the Scientific Medical Societies (AWMF)
► Federal Government Commissioner for the Interests of Patients
► Federal Association for Self-Help of People with Disabilities and Chronic Illness and their Relatives (BAG SELBSTHILFE e.V.)
► German Medical Association
► Federal Ministry of Labour and Social Affairs
► Federal Ministry of Education and Research
► Federal Ministry for Family Affairs, Senior Citizens, Women and Youth
► Federal Ministry of Health
► Federal Chamber of Psychotherapists
► Federal Association of the Pharmaceutical Industry (BPI)
► Federal Association of Medical Technology
► German Dental Association
► German Research Foundation (DFG)
► German Association of General Practitioners
► German Hospital Association
► German Nursing Council
► Eva Luise and Horst Köhler Foundation for People with Rare Diseases
► Federal Joint Committee
► Umbrella Association of the Statutory Health Insurance Funds
► Federal Association of Statutory Health Insurance Physicians
► Federal Association of Statutory Health Insurance Dentists
► Medical Faculty Association of the Federal Republic of Germany (MFT)
► Orphanet-Germany
► Association of Private Health Insurance Funds
► Association of Research-Based Pharmaceutical Companies – vfa bio
► Association of University Hospitals in Germany (VUD)
► Association of the Diagnostics Industry (VDGH)

## 2. Developments in the provision of care to people with rare diseases

### Care centres for rare diseases

**Figure 2:**
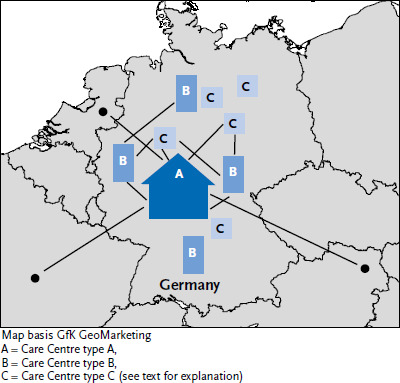
Interconnected centre model according to NAMSE Source: own illustration

NAMSE recommended the introduction of an interconnected centre model in the ‘Care, Centres, Networks’ field of action of the National Action Plan [7]. This is based on three interconnected levels, which are structured according to the division of labour and which should not differ in terms of the quality of the care they provide, but in the range of services they offer. This centre model is designed to promote cooperation between the respective specialists as well as the sharing of expertise in the field of rare diseases on both a national and an international level. This is intended to establish the prerequisites for the provision of care close to home (Figure 2). Above all, the latter is also a demand of the patient self-help groups.

The type A centres, reference centres for rare diseases, are in charge of both inpatient and outpatient care and are the point of contact for patients with an unclear diagnosis. They have guides and capabilities for interdisciplinary case conferences and innovative special diagnostics. Type B centres, specialist centres for a specific disease or group of diseases, also work on an outpatient and inpatient basis, but are in charge of the provision of care to people with a confirmed diagnosis or a solidly suspected diagnosis, e.g. a centre for rare neurological diseases. These disease-specific centres of expertise form the building block of the European Reference Networks (ERNs) required by Directive (2011/24/EU) [2] on the application of patients’ rights in cross-border health care. Type C centres provide disease or disease group-specific outpatient care in an interdisciplinary and multiprofessional setting. A type C centre is primarily in charge of providing specific care services to patients with a confirmed diagnosis or a clear suspected diagnosis. The implementation of this network model is the central requirement at the heart of the National Action Plan of 2013. NAMSE has formulated quality criteria for structures and processes in the centres, which have been applied since 2021 through the involvement of an independent certification body within the framework of a certification procedure for type A centres [8]. A certification procedure for type B centres is in preparation.

**Figure 3:**
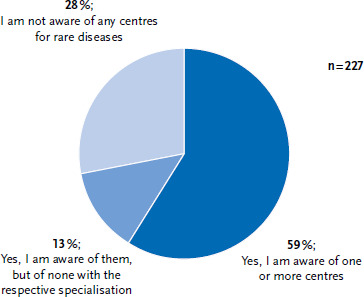
Awareness of the existence of the Centres for Rare Diseases Source: online survey of afflicted individuals as part of the expert report on the health status of people with rare diseases in Germany [6]

More than 30 centres for rare diseases have been established at university hospitals in Germany in recent years (for an overview, see www.se-atlas.de). Some of the facilities have already successfully completed the certification procedure. They demonstrated that they meet the quality criteria formulated by NAMSE. The certification procedure is designed to promote transparency of the care scenario for patients, relatives and physicians in private practice. Although specialised centres have been established according to these criteria in recent years, an online survey conducted for the 2023 report on the health status of people with rare diseases indicates that those afflicted are not yet sufficiently aware of these structures (Figure 3).

**Infobox 3 Fields of action of the National Action Plan**

The National Action Plan for People with Rare Diseases proposes measures in the following fields of action:

► Care, centres, networks
► Research
► Diagnosis
► Registry
► Information management
► Patient orientation

### Testing new care approaches: Innovation Fund Projects TRANSLATE-NAMSE and ZSE-DUO

The TRANSLATE-NAMSE project was funded from 2017 to 2020 in the framework of the Innovation Fund at the Federal Joint Committee (G-BA) aiming to introduce and solidly implement the centre structures and processes proposed by NAMSE. Guides and medical coordinators, an essential element of the structural quality of a NAMSE type A centre, were recruited at all participating sites as part of the project. A total of ten university hospitals with outstanding clinical expertise were involved and were linked within the framework of process quality. Some of the centres in this network also took on innovative special diagnostics. Interdisciplinary case conferences were the main building block and a new service in the context of which the need for this diagnostic work-up was affirmed. The new forms of care tested in the project significantly shortened the diagnostic process and significantly improved the efficiency of the care provided. For example, the diagnostic process for patients without a confirmed diagnosis took just half a year, while children had previously been treated for their symptoms in various care facilities for an average of four years and adults for eight.

Still, it became clear that there is still a low level of awareness of the existence of the care services offered by type A Centres among primary care providers and patients [9].

The ZSE-DUO project (Dual guide structure for clarifying unclear diagnoses in centres for rare diseases) [10], funded by the Innovation Fund, also proposes suitable disease-transcending structures and processes in order to identify diagnoses for people with unclear diagnoses and suspected rare diseases. Here, a dual guide contact point was created that involves not only a somatic specialist, but also psychiatric-psychosomatic expertise. This is intended to reduce the time to diagnosis and care offers are to be made available to the patients more readily and efficiently. As such, the project addresses the need for psychological support, which was once again highlighted in the current report of the Fraunhofer Institute for Systems and Innovation Research IS [6], although access to and the ensuing utilisation of psychosocial care services is still considered to be rather difficult by some of the stakeholders surveyed in the report. The lack of psychotherapists is seen as the root cause in this regard. Self-help continues to play a major role in the support for patients at this point, in that it facilitates discussion offers of a peer-to-peer type of sharing and by providing knowledge and information that is made comprehensible for laypersons. Patient self-help plays an important role specifically in the field of rare diseases, because patients and their relatives are actually the experts for the respective disease and are therefore important partners for those attending to them.

The Alliance for Chronic Rare Diseases – or ACHSE for short – is the umbrella organisation of and for people with chronic rare diseases and their relatives in Germany. Comprising 130 patient organisations, ACHSE pools the existing expertise and knowledge in the field of rare diseases and represents the interests of the afflicted people.

### Legislation for better care for rare diseases

Some ten years after publication of the National Action Plan, NAMSE has initiated important improvements in the field of care [11]. The Federal Joint Committee (G-BA) defined the special tasks of centres for rare diseases and established nationwide quality requirements for the first time at the end of 2019 in the Act to Strengthen Nursing Staff (Nursing Staff Strengthening Act), § 136c section 5 of the Fifth Social Code (SGB V) [12]. This was taking into account the requirements for centres previously defined by NAMSE. These special tasks are to be financed through centre surcharges. This is the prerequisite for a sustainable implementation of the centre model.

With the 2015 Patient Data Protection Act (PDSG), a semantics centre was established at the Federal Institute for Drugs and Medical Devices (BfArM), and the Digital Care and Nursing Modernisation Act (DVPMG) of 3 June 2021 established the basis for specification of the unique coding of rare diseases in inpatient settings. Implementation has been mandatory since 1 January 2023. An important milestone has been reached through the mandatory, precise coding of rare diseases. Coding makes rare diseases visible. The precise, digitally evaluable designation of the diagnosis is also relevant not only for billing purposes, but also in particular for epidemiology, research and the application of artificial intelligence (AI). It is also of elementary importance for patient safety, so that in the clinical context – including, and especially, in emergencies – special risk factors and therapeutic needs can be taken into account immediately [13].

Many other projects and (legal) initiatives have been initiated in recent years and contribute to the improvements in the current care scenario. For an overview of selected projects and initiatives, see Table 1.

## 3. Discussion and conclusion

Many important milestones in the provision of care have been achieved since the National Action League for People with Rare Diseases was founded in 2010.

The expert report on the health status of people with rare diseases [6], commissioned by the BMG and published in 2023, states that the care status of people with rare diseases has improved compared to 2009 and to the introduction and implementation of the National Action Plan (NAP) in 2012. In particular, the establishment of a specialised and interconnected care structure through the development and establishment of the recommended centre structure involving type A and type B centres is seen as central in this context. More detailed planning of the type C centres for quality-assured care close to home is still pending. Points of contact for people with rare diseases have been established through the introduction of these specialised structures. The effectiveness of these new approaches to networked care was demonstrated and established locally in the TRANSLATE-NAMSE and ZSE-DUO innovation fund projects [9, 10]. Permanent adoption of the new services through their inclusion in the standard care system is an important next step. The new financing instruments introduced in the form of the centre surcharges strengthen this network structure.

The survey of afflicted people within the framework of the expert report showed that almost two thirds of respondents had noticed improvements in medical care over the past 10 to 15 years, but that little improvement was noticeable with regard to social participation and the effects of the disease on their economic status and their overall living conditions [6].

According to the report [6], the availability of drugs, another important area of care for people with rare diseases, is rated as being good by the stakeholder groups surveyed. The main underlying reason being that new medicines are made available immediately after approval in Germany. According to the survey, orphan drugs (drugs for rare diseases) provide new treatment options for some people with rare diseases; the number of available preparations is said to have increased in recent years. At the same time, however, it was pointed out that there are still no therapies available for many rare diseases and that the availability of drugs for these patients remains unchanged. Thus, many afflicted persons are (still) dependent on offlabel use, i.e. the use of a drug outside its areas of application as approved by the regulatory authorities. However, there is no indication from the interviews of any problems occurring particularly frequently in this regard.

The uniform and unambiguous coding in the inpatient setting by means of Alpha-ID-SE, which will become mandatory on 1 January 2023, will for the first time enable unambiguous recording and thus better visibility of rare diseases for the purposes of rare disease research and care. The National Registry for Rare Diseases (NARSE) [14] initiated by the Eva Luise and Horst Köhler Foundation is designed to systematically record especially very rare diseases in order to improve the respective care in the future. The effectiveness of this registry is to be tested within the framework of the FAIR4Rare innovation fund project [15].

Improvements, especially in the diagnosis of rare diseases, might also be on the horizon due to the use of artificial intelligence, although there are still many open questions to be discussed and answered [6]. Undoubtedly, the various projects and political undertakings set up important prerequisites in recent years with regard to the harmonisation of data, their consolidation and use, which form an important basis for the application of AI.

According to the above-mentioned expert report, challenges exist in the connection of the centres in particular to SHI-accredited medical care [6]. Although the introduction of the medical care atlas for rare diseases (se-atlas) in 2015 established an overview of the existing care structures, these still seem to be known only to an insufficient extent by the primary care providers. According to the expert opinion, a good and low-threshold offer is available according to the centres’ point of view, which, however, obviously does not reach the target group. Whether or not the afflicted receive a specific diagnosis and ensuing treatment or continue to go through an odyssey still seems to depend largely on coincidence and their individual commitment. The BMG-funded project ‘Evaluation of interface management concepts for rare diseases: systematic stocktaking & development of best practice recommendations (ESE-best)’ [16] from 2022 also concluded that the care landscape has changed for the better through the introduction of the centre model, but that this specialised care is characterised in particular by interface problems that can significantly impair the quality of care. Among other things, deficits in communication and information transfer between the sectors and organisational deficits are mentioned here. The project has formulated recommendations for the management of interfaces. The BMG has commissioned the NAMSE office to work towards implementation of the recommendations in health care practice. The NAMSE working groups will address these topics in 2023 to 2026 and develop proposals for solutions.

In conclusion, it can be said that effective building blocks for improving the care provided to people with rare diseases have been developed since the National Action Plan was published in 2013, and must now be linked together in order to achieve an improvement in care as the dedicated goal of the initiative. The fact that people with more common diseases also benefit from further development of health care for rare diseases is also emphasised in the conclusion of the report: ‘Rare diseases are some sort of ‘burning lens’, as it were, for the German health and social care system and allow fundamental challenges and needs for further development to come to the fore at an early stage. As such, rare diseases are an important opportunity allowing us to draw lessons for further development of the health care system and for identification of best-practice approaches from which the provision of care could benefit across the board. Accordingly, rare diseases can help to ensure that all people, regardless of the prevalence of their individual condition, receive good, needs-based health care and are enabled to participate in society.’ [6, p. 64]

## Key statement

- Rare diseases require a specialised type of care.
- The National Action League for People with Rare Diseases recommends a three-tier centre model in the National Action Plan.
- Research projects supported by the Innovation Fund demonstrate the effectiveness of the structure comprising three types of care centres.
- Care has improved steadily since 2009, notes the expert report on the health status of people with rare diseases of the Fraunhofer Institute for Systems and Innovation Research ISI from 2023.
- There is a need to implement structured patient pathways to further develop the provision of care.

## Figures and Tables

**Table 1:** Expert opinions, recommendations and projects related to rare diseases Source: own illustration

Study	Topic/goals	Time	Source
**Expert reports, recommendations and action plans**			
Study on the health status of people with rare diseases	Measures to improve the health status of people afflicted by rare diseases	2009	[3]
EU Recommendation for Action in the Field of Rare Diseases	Recommendation on diagnosis, treatment and care for people afflicted by rare diseases	2009	[2]
National Action Plan for People with Rare Diseases		2010	[7]
Expert opinion of the Fraunhofer Institute for Systems and Innovation Research (ISI)	Health status of people afflicted by rare diseases	2023	[6]
**Research**			
WB-NAPSE	Scientific monitoring of the National Action Plan	2019	[5]
TRANSLATE-NAMSE	Implementation of the goals of the National Action Plan in the area of health care	2017 – 2020	[9]
ZSE-DUO	Dual guide structure for clarifying unclear diagnoses in centres for rare diseases	2018 – 2022	[11]
NARSE – National Registry for Rare Diseases	Establishment of a national registry for rare diseases	since 2021	[14]
FAIR4Rare	Accompanying evaluation of the set-up process of an open National Registry for Rare Diseases (NARSE)	2023 – 2025	[15]
ESE-Best	Evaluation of interface management concepts for rare diseases	2019 – 2022	[16]
**Miscellaneous**			
se-atlas	Care atlas for people with rare diseases	since 2015	[17]
Centre surcharges	Hospitals acting as centres that perform special inpatient tasks can receive financial surcharges for this purpose	2019	[12]
Certification procedure for centres for rare diseases	Certification by independent certification body clarcert GmbH	2021	[8]
